# IARC 2019: “Night shift work” is probably carcinogenic: What about disturbed chronobiology in all walks of life?

**DOI:** 10.1186/s12995-019-0249-6

**Published:** 2019-11-27

**Authors:** Thomas C. Erren, Peter Morfeld, J. Valérie Groß, Ursula Wild, Philip Lewis

**Affiliations:** Institute and Policlinic for Occupational Medicine, Environmental Medicine and Prevention Research, University Hospital of Cologne, Cologne, Germany

**Keywords:** Night work, Shift work, Circadian, Chronodisruption, Cancer, Breast, Prostate, Internal time

## Abstract

In June of 2019, a working group convened by the International Agency for Research on Cancer [IARC] concluded that “night shift work” is probably carcinogenic to humans (a Group 2A carcinogen). This was based on sufficient evidence of cancer and strong mechanistic evidence in experimental animals and limited evidence from human epidemiological studies. The biological basis from experimental work is clear and compelling: Disturbed chronobiology such as due to alterations in the light-dark schedule which shift-workers experience is associated with carcinogenicity. But is it correct to assume in epidemiological studies that “night shift work” provides the same dose of disturbed chronobiology to all night workers and that disturbed chronobiology from activities outside of work does not count? Both chronobiological theory and supporting evidence suggest that much-needed future epidemiology should address these questions and should consider disturbed chronobiology in all walks of life.

Epidemiology is certainly a poor tool for learning about the mechanism by which a disease is produced, but it has the tremendous advantage that it focuses on the diseases and the deaths that actually occur, and experience has shown that it continues to be second to none as a means of discovering links in the chain of causation that are capable of being broken.

-Sir Richard Doll [1].

## Background

In June 2019, a working group convened by the International Agency for Research on Cancer (IARC) concluded that “night shift work” is “probably carcinogenic to humans” (Group 2A) [2]. Clearly, the 27 experts from 16 countries weighed today’s evidence appropriately. Equally clearly, however, a key question for coming years is whether more chronobiology-targeted epidemiology with the scope broadened to include both occupational + environmental settings may provide different evidence to arrive at a more concrete conclusion.

The working group importantly qualified what was evaluated as comparatively different to IARC’s 2007 verdict. That is to say, IARC classified “shiftwork involving circadian disruption” as probably carcinogenic to humans in 2007 [3] and this year the working group zoned in on “night shift work” [2]. The rationale is that night shift work better reflects the main evidence base for the human cancer studies [2]. This exposure focus was in line with what Travis et al. described in 2017: “Our aim was specifically to examine the hypothesis that night shift work, and particularly long-term night shift work, increased breast cancer risk” [4].

Remarkably, what was concluded from experimental evidence could potentially suffice for a Group 1 classification: Regarding the carcinogenicity of alternating light-dark schedules and key characteristics of carcinogens, the working group found sufficient evidence of cancer and strong mechanistic evidence in experimental animals [5]. Insofar, this situation is contrary to what epidemiologists regularly experience. In fact, epidemiologists often quest biological plausibility as a key viewpoint to decide whether (or not) to pass from observed statistical associations to a verdict of causation. In the carcinogenicity of shift work case, (chrono-)biological plausibility is clear-cut but epidemiology does not deliver a similar verdict – or at least just not yet.

### Capturing disturbed chronobiology

With continued Group 2A classification after 12 years of extensive research, note that “limited evidence of cancer in humans” was the weakest stream of evidence when evaluating carcinogenicity in both 2007 and 2019 [2, 3]. As humans are no big rats, epidemiology is indispensable for judging whether what experiments suggest also pertains to the real world of humans. In this case, to arrive at a concrete conclusion of the exposure to (night) shift work being carcinogenic to humans or not, epidemiology must focus on what experiments suggest as “causal”; namely, disturbed chronobiology. To capture disturbed chronobiology – be it described as misalignment or disruption of circadian rhythms of normal physiology [2], chronodisruption, circadian misalignment or other [6] – epidemiology must utilise appropriate dose metrics.

Why is this important and how can we achieve this? Repeatedly, it has been pointed out that information on chronotype (an individual’s preferential timing for being awake and asleep) [7, 8] – acting as an effect modifier – and the assessment of doses may be a must to study relationships between disturbed chronobiology and cancer [9, 10]. Epistemologically, shift work epidemiology to-date may have misclassified doses (potentially even exposures) in two ways. First, individual internal time architecture may determine different doses of disturbed chronobiology by a given shift. Thus, counting shifts alone – be they night or other shifts – without relating them to internal time cannot suffice. Second, doses of disturbed chronobiology can be expected whenever we live against our endogenous clocks [11]. For instance, light [2, 12] or activities [13] when our bodies expect sleep may be associated with disturbed chronobiology in non-work settings also. As such, focusing on disturbed chronobiology at workplaces alone could be analogous to 1940s smoking research had it focused on smoking in workplaces alone (i.e. result in a misclassification) [14].

Of course, we appreciate that capturing doses accumulated in occupational + environmental settings will be difficult but it will be necessary to examine health effects plausibly (co-)determined by disturbed chronobiology over extended periods of time. In principle, information on how much time individuals sleep during their biological day or are active during their biological night allows to calculate doses of disturbed chronobiology.

Let us exemplify how this could be done: The German National Cohort (GNC), a prospective cohort study could provide the basis for such chronobiology-targeted epidemiology [15]. The GNC follows random samples of 200,000 women and men aged 20–69 years from the general population over 25 to 30 years. Recurrent questions regarding sleep timing can allow determination of both chronotype and disturbed chronobiology [6, 11]. Thus, GNC sleep details should allow epidemiological exploration of chronobiology-targeted risk predictions such as e.g. increased doses of disturbed chronobiology are associated with increased cancer risk. Such prospective studies would be the gold standard to explore dose-relationships between disturbed chronobiology accumulated in occupational + environmental settings and cancer; they shall need lengthy follow-up, though. Case–control studies requiring information on study individuals’ chronotype and time windows of sleep or activities over time will be more challenging. Difficulty determining chronotype applies to retrospective industry-based cohort studies as well. Yet, that the latter offer documented work times may allow inferring activity and sleep times over decades-of-interest. Finally, chronobiology-targeted analysis strategies may also inform how we collect chronotype and working and/or sleep time information [16], preferably in time-dependent manners [17].

A few cohort and case-control studies have considered chronotype in their assessments but found little-to-no-effect on breast or prostate cancer outcomes [18–23], although there may be some suggestion of increased evening type susceptibility [20, 21]. On the face of it, this seems counter-intuitive; however, such conclusion would be imprudent for two reasons: First, epidemiologists must include information on chronotype as an effect modifier; second, disturbance to chronobiology outside the workplace was not accounted for in these studies. That the few studies who included chronotype assessment found risk differences, regardless of whether it is in line with what we predict, highlights that such information can impact findings and needs to be considered.

The more we study effects of disturbed chronobiology the more we appreciate the complexity of how species, including man, organize physiology over biological days and nights as an evolutionary legacy in response to light/dark transitions of our planet. Interpretable epidemiology must at least begin to consider this complexity.

## Conclusion

Overall, we can’t put our conclusions too strongly: Likely ubiquitous exposures to disturbed chronobiology will make interpretable epidemiology one big challenge. However, while the outlined research faces open questions – e.g. how to ‘best’ assess internal time [6] – future ‘epidemiology-as-usual’ will likely confront us with inconsistent results similar to those identified by IARC experts just now. The world’s leading authority on carcinogens has declared 1 in 5 workers worldwide exposed to a “probable” carcinogen. Disconcertingly, the pervasive potential of disturbed chronobiology in occupational + environmental settings could imply that this candidate cause of cancer may yield numerous cases with substantial effects, thus contributing significantly to the population burden. A much-needed next wave of epidemiological studies into effects of disturbed chronobiology and cancer must provide *interpretable* information as to whether ‘working and living against endogenous clocks’ is carcinogenic [11].
